# Evaluation of the Impact of Immunization Second Year of Life Training Interventions on Health Care Workers in Ghana

**DOI:** 10.9745/GHSP-D-21-00091

**Published:** 2021-09-30

**Authors:** Dieula Delissaint Tchoualeu, Bonnie Harvey, Mawuli Nyaku, Joseph Opare, Denise Traicoff, George Bonsu, Pamela Quaye, Hardeep S. Sandhu

**Affiliations:** a Global Immunization Division, Center for Global Health, U.S. Centers for Disease Control and Prevention, Atlanta, GA, USA.; b African Field Epidemiology Network, Accra, Ghana.; c Ghana Health Service, Public Health Division, Disease Control and Prevention Department, Expanded Programme on Immunization, Korle Bu, Accra, Ghana.

## Abstract

**Introduction:**

As part of a suite of training interventions to improve the knowledge and practice of immunization in the second year of life (2YL), training of trainers workshops were conducted with regional and district health management teams (DHMTs) in 15 districts in 3 regions of Ghana. Using adult learning principles, DHMTs implemented several capacity-building activities at the subdistrict and health facility levels, including health facility visits, on-the-job training, and review meetings. The current evaluation investigated whether frontline health care workers (HCWs) reported or demonstrated improvements in knowledge, attitudes, and practices after training interventions.

**Methods:**

Quantitative and qualitative methods with a utilization-focused approach guided the framework for this evaluation. A systematic random sample of 115 HCWs in 3 regions of Ghana was selected to complete a competency survey before and after training, which focused on 3 core competency areas—Expanded Programme on Immunization (EPI) policy; communication with caregivers; and immunization data management, recording, and use. Interviews and direct observations by data collectors were done to assess HCWs’ knowledge, self-reported attitude, and behavior changes in practices.

**Results:**

Of 115 HCWs, 102 were surveyed before and 4 months after receiving capacity-building interventions. Modest but not statistically significant improvements were found in knowledge on EPI policy, immunization data management, and communication skills with caregivers. HCWs reported that they had improved several attitudes and practices after the 2YL training. The most improved practice reported by HCWs and observed in all 3 regions was the creation of a defaulter list.

**Discussion:**

Findings of this evaluation provide encouraging evidence in taking the first step toward improving HCW knowledge, attitudes, and practices for 3 core immunization competency areas. The use of learner-focused teaching methods combined with adult learning principles is helpful in solving specific performance problems (such as lack of knowledge of EPI policy).

## Abstract (precis)

Applying performance-based training interventions that follow adult learning principles and include follow-up activities after training may help to solve specific performance problems and improve health care workers’ performance in immunization service delivery. These strategies facilitate learning, minimize the forgetting curve for health care workers, and should be considered as a standard practice for future training interventions.

## Teaser

**Key Findings:**

- After implementing the immunization Second Year of Life capacity-building interventions, quantitative and qualitative data showed that health care workers (HCWs) reported a modest improvement in knowledge, attitudes, and practices in 3 performance problem areas: lack of knowledge on EPI policy; inconsistent data management, quality, and use; and weak communication about vaccination with caregivers.
- Trainers in district health management teams used a variety of teaching methods and delivery techniques to facilitate HCWs’ learning and help minimize their forgetting curve.

**Key Implications:**

- National and subnational leaders should take ownership of the capacity-building needs of their immunization workers and leverage existing mechanisms, such as new hire orientation, supportive supervision visits, and monthly data review meetings to empower their health care workers to perform EPI tasks more proficiently.
- Countries should value budgeting for capacity building of their immunization workforce and for behavior change evaluation to ensure an accurate understanding of capacity-building impact.
- Stakeholders should define their expectations of specific tangible outputs from training that link to a measurable performance objective, instead of broad “refresher” training.

## INTRODUCTION

The Second Year of Life (2YL) platform promotes vaccinating children aged 12 to 24 months and beyond with recommended vaccines to reduce morbidity and mortality from vaccine-preventable diseases by increasing population immunity during childhood and contributes to disease control and elimination goals.^1^^,^^2^ The 2YL vaccination platform can serve as a catch-up opportunity for children who have missed vaccine doses during the first year of life and enables the administration of booster doses and underutilized or newly introduced vaccines given during 2YL.^2^ It can also facilitate health system strengthening via integration of immunization services with other preventative health interventions and programs (e.g., deworming, growth monitoring, and bed net distribution) during the post-infancy period.

The World Health Organization (WHO) recommends a second dose of measles-containing vaccine (MCV2) in the second year of life.^3^ WHO’s Global Measles and Rubella Strategic Plan 2012–2020 set a milestone of attaining 95% coverage by 2020.^4^ High MCV2 coverage is essential to achieving herd immunity within a country and achieving global measles elimination goals. Ghana introduced MCV2 at the 18-month child wellness visit in 2012 as part of their national immunization schedule. During the same year, Ghana also added 2 new vaccines to be given during the first year of life, 2 doses of rotavirus vaccine (6 and 10 weeks) and 3 doses of pneumococcal conjugate vaccine (PCV) at 6, 10, and 14 weeks.^5^^,^^6^ In 2015, 3 years after introduction, MCV2 coverage was just 63%, while coverage with the last doses of PCV and rotavirus vaccine had reached 88%.^5^ The United States Centers for Disease Control and Prevention (CDC) collaborated with Ghana Health Service (GHS) in 2015 to implement multifaceted 2YL interventions. Ghana was selected because of its leadership in the African region in expanding routine vaccination to the 2YL, its low MCV2 coverage and variation in immunization coverage across districts, and its plan to introduce meningococcal A conjugate vaccine (Men A) in 2016. Ghana was the first country in the African region to provide this vaccine during 2YL in the routine immunization schedule.

In mid-2016, CDC and GHS conducted baseline health facility and household surveys in 3 underperforming regions of the country to understand factors associated with poor MCV2 coverage.^5^ Survey results indicated that a 9-year absence of EPI staff training, inconsistent supportive supervision and defaulter tracing, weak communication between health care workers (HCWs) and caregivers, and poor documentation of data contributed to the low MCV2 coverage. Additionally, findings revealed coverage inequities across the population for a variety of antigens among districts in the regions surveyed.

In mid-2016, CDC and GHS conducted baseline health facility and household surveys to understand factors associated with poor MCV2 coverage.

Based on the 2016 survey results, CDC recommended that GHS implement various practical strategies (e.g., improving the number, content, and quality of supportive supervision visits, on-the-job training at health facility level) to improve program performance at subdistricts and health facilities in these underperforming regions. GHS requested CDC to provide technical and financial support on 3 training of trainers (TOTs) workshops for district health management teams (DHMTs). Both institutions worked closely to design, implement, and evaluate these interventions. (Details of these TOTs are provided in the Methods section.) Trained DHMTs then implemented a variety of capacity-building interventions to transfer knowledge and skills related to 2YL immunization to frontline HCWs during the same year. This article describes an evaluation conducted to answer 2 questions:

1. Did frontline HCWs’ knowledge of EPI policy, immunization data management and use, and communication with caregivers increase after the DHMTs’ interventions?
2. How did frontline HCWs’ attitudes and practices regarding 2YL vaccination change after receiving the DHMTs’ capacity-building interventions?

## METHODS

### Intervention Overview

GHS and CDC conducted TOT workshops from July through September 2017 in 3 of the most underperforming regions with respect to low MCV2 coverage: Greater Accra Region (GAR), Volta Region, and Northern Region (NR). The TOTs targeted regional health management teams (RHMT) and DHMTs in a total of 15 districts—2 high-performing and 3 low-performing teams per region, based on selected EPI indicators (i.e., coverage of third dose of pentavalent vaccine [Penta 3], first dose of measles and rubella vaccine [MR1], and second dose of measles and rubella vaccine [MR2], and dropout rates). Thirteen of the 15 districts were urban, and the other 2 were both urban and metropolitan (1 in GAR and 1 in NR). In total, the TOTs trained 74 health professionals—24 in GAR, 22 in Volta, and 28 in NR.

The objectives of the TOTs were to improve knowledge and skills at the district and regional levels related to immunization for children in their 2YL and to strengthen participants’ ability to train the frontline HCWs in their districts. As described by Traicoff et al.,^7^ these TOTs covered adult learning principles (including classroom management), technical (e.g., EPI policies, measles immunogenicity, defaulter tracing), and operational (e.g., best practices of supportive supervision, problem analysis, and prioritization) topics. The authors described in detail the TOTs curriculum design and the activities conducted during and after implementation of the TOTs. DHMTs also received specific guidance on how to effectively plan, implement, and evaluate capacity-building interventions. DHMTs in turn were instructed to transfer their skills and knowledge to frontline HCWs at the subdistrict and health facility levels by implementing several capacity-building interventions, including workshops, health facility visits, on-the-job training, and review meetings. The RHMTs were expected to provide leadership oversight, technical guidance, and support to the DHMTs during the implementation of these interventions.

### Evaluation Approach

Patton’s^8^ utilization-focused evaluation approach was applied to maximize the use of evaluation findings for improving workforce performance. This evaluation method uses a participatory approach through which evaluators and intended users come together to design and implement the evaluation, analyze the data, and collaboratively review the results to increase the utilization of the findings to improve performance. GHS and CDC staff co-designed an evaluation, met after piloting to rework the data collection tools, and reviewed the results in a moderated discussion. The quantitative component of the evaluation consisted of a survey to assess knowledge of frontline HCWs before and after capacity-building interventions. This component also included a DHMT activity tool to document the scope of their field activities and specific methods/strategies used to deliver the interventions. The competency scaling was developed based on the National Institutes of Health’s Proficiency Scale and adjusted to meet the expected competencies of the HCWs within the 3 main training topics (EPI policies, data management, and communication).^9^ The qualitative components consisted of interviews with HCWs (i.e., subdistrict/health facility level immunization staff) and participant observations to understand their self-reported behavior changes and to receive feedback on the quality and delivery of the interventions.

A utilization-focused evaluation approach was applied to maximize use of evaluation findings for improving workforce performance.

### Sample

For each of the 3 participating regions in this evaluation, the total number of targeted frontline HCWs who required interventions varied based on several characteristics as defined by the DHMTs—length of time working in health care, age, location, and the likelihood of continued years in health care. To be eligible for both intervention and evaluation, HCWs had to be working in 1 of the 15 districts that hosted the TOT workshops. In addition, they had to either be (1) subdistrict HCWs working on immunization services 5 years or less, or (2) newly hired HCWs (in the last 5 years) who did not receive a new hire orientation on immunization services. HCWs planning to retire within the next 5 years were excluded. Based on these criteria, 1,310 HCWs were eligible for training (GAR, 513; Volta, 336; NR, 461) in 15 districts within the 3 regions. Study investigators consulted with a statistician to construct a sample frame from the number of eligible participants. A systematic random sample of 7 HCWs for each of the 15 participating districts was selected from the list of eligible participants, except for in Accra Metro Sub-Metro (equivalent to a district) in GAR. An additional 10 HCWs were added to the GAR sample to account for the much larger number of health facilities in the Accra Metro Sub-Metro areas compared with the other subdistricts. Therefore, 17 HCWs were selected for this district, for a total of 115 HCWs across all 3 regions.

### Data Collection Instruments

A simple DHMT activity report in ODK was developed for the project and was used by the district staff to report for each HCW capacity-building field activity in their respective region, the specific methods/strategies used to facilitate learning, and the topics they taught during each session. The DHMTs submitted this report every time they conducted training or shared knowledge on the 3 core competencies that were covered during the TOT.

We designed a progress analysis survey (PAS) that included quantitative and qualitative closed and open-ended questions aimed at answering the 2 evaluation questions. The questions were designed based on the training curriculum used in the TOT workshops (which was expected to then be adapted for the post-TOT HCW interventions). The PAS included core competencies questions, basic demographic information (age, sex, years in service, and job title), and the date and format of the last 2YL intervention they had received. The questions focused on 3 core competency areas of the TOT curriculum.

- EPI policy: Standard operating procedures around routine and catch-up vaccination, simultaneous injections (i.e., vaccination conducted during the same session but different site), decision-making algorithm for when to administer MCV2 or Men A, intervals between vaccines, preventing missed opportunities for immunization, and how to manage adverse events following immunization
- Immunization data management, quality, and use: Accurately documenting data in tally books, child welfare clinic (CWC) registers, child health record booklets, and monthly vaccination reports; updating monitoring charts; and defaulter identification process for defaulter tracing
- Communication: Key immunization messages for caregivers (increasing parents’ awareness of MCV2 and Men A), including what to expect from a 2YL visit, importance of 2YL vaccines, and addressing vaccine hesitancy

GHS EPI experts reviewed the PAS for technical accuracy before piloting it at a health center in GAR. Data collectors practiced with health center staff at the pilot side while one of the primary investigators observed, and then the team (CDC, GHS EPI experts, and data collectors) revised the PAS again for content, clarity of questions, and format. Next, GHS experts validated the revised tool, reviewing it for accuracy on technical content and optimizing it for local context before data collection. Quantitative responses were scored on a Likert scale, adapted from National Institutes of Health’s Proficiency Scale,^9^ as follows:

1 = No knowledge. Participant is unable to say anything about the skill or demonstrate any ability to perform the tasks associated with the skill.

2 = Novice (basic knowledge). Participant has a rudimentary understanding of basic techniques and concepts.

3 = Intermediate (practical application). Participant can successfully complete tasks or answer questions in this competency as requested. Help from an expert may be required from time to time, but the participant can usually perform the skills independently.

4 = Advanced (applied theory). Participant can perform the actions associated with this skill without assistance. They can provide guidance, troubleshoot, and answer questions related to this area of expertise and the field where the skill is used.

The data collectors also used the PAS to record observations to understand if and how attitudes and practices around 2YL services had changed. Key observations included participants’ knowledge on how to use the CWC register, the decision-making algorithm for when to administer MCV2 or Men A, availability of data collection reporting tools, presence (or lack of) a monitoring chart at the health facilities, and any changes observed in practice during PAS2. All the observations were noted and recorded in ODK.

### Data Collection

Data collection occurred in 2 phases. The first progress analysis survey (PAS1) was conducted after the TOT workshops (before DHMTs trained HCWs) during November 2017, and the second progress analysis survey (PAS2) occurred during March 2018 after DHMTs implemented a variety of capacity-building interventions with HCWs. The time between PAS1 and PAS2 provided the HCW training participants a chance to practice what they learned during the training. Both PAS1 and PAS2 were administered by trained data collectors and used at both points to assess knowledge of the 3 core competencies. For PAS2, a few questions were added to understand the format and date of the 2YL intervention(s) HCWs recently received, their opinions on the intervention(s), and their self-reported behavior change.

Data collection occurred in 2 phases, and the time between phases allowed HCW training participants a chance to practice what they learned in training.

During both surveys, data collectors noted key observations within a structured observation framework while at each collection site, but more extensive field notes about progress or changes since the first visit were encouraged for the second phase. GHS and CDC contracted the Ghana Field Epidemiology and Laboratory Training Program (GFELTP) to collect and analyze the data for PAS1 and PAS2. GFELTP affiliates served as data collectors and received training on the following areas: Android tablet use, how to navigate the LINKS app (i.e., the app used for data collection) and download forms, the basics in Ghana EPI competency standards, key evaluation research questions, and administration of the tool. In addition, data collectors received guidance on the scoring system and definition of key variables. The data collectors were paired up to visit each health facility and administer the survey. They were also assigned to the same sites for both PAS1 and PAS2 to facilitate better observation of behavior and knowledge change. The data collectors were instructed with specific key changes to note during PAS2 data collection (e.g., was the monitoring chart updated, was a data review meeting conducted; observation of improvement in attitudes and practice). We took these measures to minimize potential selection, social desirability, recall, and personal biases.

An electronic version of the PAS was stored in the LINKS app on a dedicated Android tablet supported by Secure Data Kit (SDK). Data collection took 1 week per region, a total of 3 to 4 weeks for each. During data collection, GFELTP affiliates uploaded completed surveys daily to the SDK database and reported field challenges through WhatsApp messaging to ensure rapid support from CDC and GHS staff who assisted with any technical or content-related questions.

### Data Management and Analysis

Collected data were stored on a cloud server; SDK form submissions were added into a database that was continuously backed up on a hard drive in a rolling 7-day window. CDC and GHS staff reviewed the data daily during active data entry/collection phases. Outliers or questionable survey entries were addressed through conversations with GFELTP and the data collectors who entered the information. CDC and GHS made all combined data available to GFELTP daily during the data entry/collection phases.

Quantitative data were stored, data quality was checked, and data were initially analyzed for descriptive purposes using Microsoft Excel. The data were then entered into Stata v. 13.0^10^ (StataCorp, College Station, TX, 2013) and a Wilcoxon signed-rank test was used to determine if knowledge changes were statistically significant in HCWs’ posttraining interventions. All qualitative data, field notes, and observations collected were uploaded into NVivo v. 12 (QSR, 2018) for analysis.^11^ Analysis included coding of field notes, observed behaviors, and practice for the expected themes as well as emergent themes. The investigators held several discussion meetings with each other and with stakeholders to review and discuss both quantitative and qualitative analyses and how they informed each other and to decide if additional analyses were needed.

### Ethical Clearance

Both CDC and GHS determined that this evaluation was a public health program activity and not human subjects’ research.

## RESULTS

### Capacity-Building Interventions Implemented After the TOT by DHMTs

Eleven of the 15 DHMTs completed activity reports that described a total of 112 capacity-building interventions conducted following the DHMT TOTs. DHMTs reported conducting workshops (n=65), health facility visits (n=43), and review meetings (n=4). Reports from the HCWs themselves in the PAS2 (described below) indicated that they also learned about 2YL via phone calls, learning from peers, and on-the-job training at the health facility level during supervisory visits.

Eleven of the 15 DHMTs completed activity reports that described a total of 112 capacity-building interventions conducted following the DHMT TOTs.

### Evaluation Question 1: Did Frontline HCWs’ Knowledge of EPI Policy, Data Management and Use, and Communication With Caregivers Increase After the DHMT Interventions?

#### PAS Survey Characteristics and Demographics

Of the 1,310 HCWs eligible for training, a total of 115 HCWs were included in the sample and all of them were surveyed in PAS1. Of these, 102 (89%) participated in at least 1 capacity-building intervention from their respective DHMT and were surveyed in PAS2. The 13 HCWs who were not surveyed in PAS2 had either not participated in any capacity-building interventions, were on leave, or data collectors were unable to locate them for the survey.

While a total of 102 HCWs were surveyed for both PAS1 and PAS2, we learned that a few DHMTs (primarily in NR) had been so enthusiastic about immediately sharing the skills and knowledge acquired in the TOT that they began implementing capacity-building interventions before PAS1 had been administered. Thus, we present the analyses only for 65 HCWs who were surveyed in PAS1 before capacity-building interventions and in PAS2 after capacity-building interventions. Data are not shown for the 37 HCWs who were included in both PAS1 and PAS2 but were surveyed in PAS1 after already being exposed to at least some capacity-building interventions. Of the total 65 HCWs surveyed at both points in time, the median age of respondents was 30 years (range 25–59 years) and a majority were women (82%). No differences were found in the distribution of demographic characteristics between the 2 groups (65 HCWs included in the analysis and 37 who were not).

#### Knowledge Growth Among the Group (n=65) First Surveyed Before Interventions

For 3 competencies evaluated in the PAS1 and PAS2 using a Likert scale rating of 1–4, there were 5 questions on EPI policy (possible score 5–20); 6 questions on immunization data management, quality, and use (possible score 6–24); and 3 questions on communication with caregivers (possible score 3–12). Of the 3 competencies, national EPI policy recorded the highest increase in knowledge with a mean score increase of 5 points (Figure a). Reviewing individual questions, the largest average increase was 1.09 points for both the catch-up policy for missed immunization and the simultaneous injection policy. Knowledge of the policy on adverse events following immunization had an average increase of 1.05 points, followed by the decision-making algorithm for when to administer MCV2 or Men A (+0.85), and intervals between doses (+0.80).

**FIGURE:**
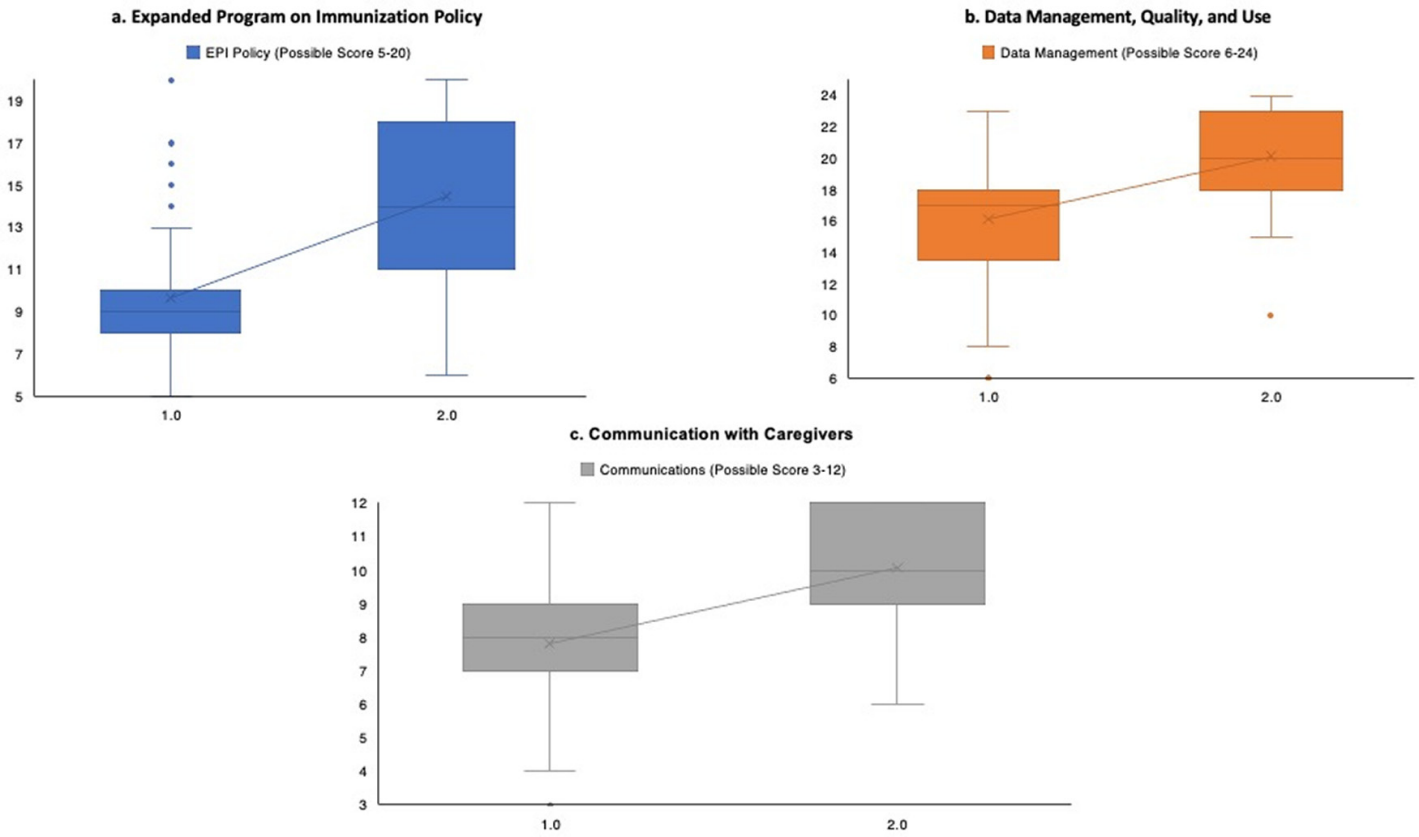
Knowledge Growth of Health Care Workers in Ghana on (a) Expanded Program on Immunization Policy; (b) Immunization Data Management, Quality, and Use; (c) and Communication With Caregivers, Before and After Capacity-Building Interventions From the First Progress Analysis Survey and the Second Progress Analysis Survey Abbreviations: EPI, Expanded Program on Immunization; PAS1, first progress analysis survey; PAS2, second progress analysis survey, PAS2.

The data management, quality, and use category averaged a mean score increase of 4.03 points (Figure b). The highest increase in the data management category was knowledge of defaulter tracing (+0.97), followed by monitoring charts (+0.83), monthly reporting (+0.71), and the CWC register (+0.65). Across all 3 competencies, the lowest increase in knowledge was in tally books (+0.42) and child health books (+0.46). No reduction in knowledge was observed between PAS1 and PAS2.

The communication with caregivers’ competency had the lowest increase in knowledge, with the mean score increasing by 2.2 points (Figure c). Individual questions on what caregivers should expect from the 2YL visit and the importance of the 2YL intervention both increased by 0.77 points, and knowledge about addressing fears and vaccine hesitancy increased by 0.71 points.

None of the demographic characteristics for the 65 HCWs (such as job designation, years of service, location, and number of job duties) were significantly linked to knowledge gained in any competency areas (data not shown). Despite the positive changes in some categories of knowledge, they were not statistically significant in a comparison of the PAS1 and PAS2 test results in all 3 areas combined (Wilcoxon signed-rank test produced a total z score of −0.772 and prob>z=0.44). We found no statistically significant differences in PAS1 and PAS2 when examining each competency score individually (data not shown).

### Evaluation Question 2: How Did the Frontline HCWs’ Attitudes and Practices Regarding 2YL Services Change After Receiving the DHMTs’ Capacity-Building Interventions?

Results from field observations and qualitative data from the PAS provide insights into behavior changes and practices of the 102 HCWs in all 3 competency areas. When the study investigators asked HCWs about their perspectives on the capacity-building activities they received, they expressed high appreciation and reported knowledge improvement after the training. For example, an HCW from the Volta region stated:

*Formerly if I met a child 17 months old I won’t give MR1 [first dose of measles/rubella vaccine], I would wait till 18 months and give MR2 [second dose of measles/rubella vaccine], but now with the help of the training, I will administer MR1 and schedule one-month interval for MR2.*

When asked about their perspectives on the capacity-building activities, HCWs expressed high appreciation and reported knowledge improvement after the training.

Similarly, another nurse from GAR reported:

*After learning about policies, I now vaccinate every child I come into contact with regardless of wastage. I have also improved on defaulters tracing.*

When asked about any changes made since 2YL training, HCWs reported that they had changed their attitudes and practices due to the 2YL training. The most improved practice reported and observed in all 3 regions was the creation of a defaulter list. For example, a public health nurse from GAR stated:

*Before the training, [I] didn't trace defaulted eligible children. But after 2YL training, I started tracing the defaulted eligible children through phone calls…*

Another HCW from NR stated:

*After the training, we realized school health can actually make a difference in our coverage. We began school health outreaches. We got about 40 children for Men A and MR2. We knew about school health but didn’t know it could be helpful.*

HCWs also reported that they changed their immunization data management practices to improve their work. The HCWs reported the importance of data validation (i.e., review data for accuracy and quality) and began to meet monthly for data validation and to check immunization coverage. The largest observed challenge was data management as HCWs across the region continued to struggle with completing reporting forms correctly and health facilities lacked the necessary data management tools (CWC registers, monitoring charts, tally books, and child health record books). When data management tools were unavailable, some HCWs improvised with personal notebooks, but several lacking the tools also struggled with the ability to do correct calculations or did not know their target population for vaccination.

HCWs also reported improvement in communication practices with caregivers during the vaccination visits due to the 2YL training. A senior nurse from NR stated:

*We now know the importance of effective communication with caregivers so we now spend more time in communicating to them to make them understand us more.*

The HCWs reported that they are more patient with the caregivers and asked their clients for feedback on their one-on-one communication during immunization sessions.

At the end of the activity tool, DHMTs were asked the following 4 open-ended questions for the 112 reported activities:

1. What were the key strengths observed during this activity?
2. What were the key challenges experienced during this activity?
3. Based on what was accomplished today, what are the next steps with this health facility?
4. Any additional comments?

The Table presents examples of key strengths and challenges observed during implemented activities reported by DHMTs.

**TABLE.** Examples of Key Strengths and Challenges Reported by District Health Management Teams in Implementing Capacity-Building Interventions to Improve Knowledge and Practice of Immunization in Ghana

**Key Strengths**	**Key Challenges**
HCWs have started school immunization.	Hard to reach communities in the zone
HCWs are conducting defaulter tracing and applying good communicating skills.	Inadequate child welfare clinic register and immunization monitoring chart; no permanent place to display chart
Various viewpoints are brought for discussion—effective participation.	Mothers reluctant to bring their children for vaccination
Good collaboration with the subdistrict is present.	Heavy workload for HCWs
Improvement in data documentation and defaulter tracing (i.e., list of children missing doses of recommended vaccines) is occurring.	Hard getting stationery from the health facility

Abbreviations: HCW, health care worker.

## DISCUSSION

Overall, findings indicate increased knowledge among frontline HCWs in all 3 competencies (EPI policy; data management, recording, and use; and communication with caregivers) in the 3 months during which DHMTs implemented a range of capacity-building interventions. Although this evaluation was not designed to assess causality or contribution of this training intervention to immunization coverage within the participating districts and regions, this was the only intervention on this subject matter in the 3 regions that was targeted to district and subdistrict HCWs within the specified time period. While knowledge improvements were not statistically significant, it is important to note that determination of the impact of an intervention should not rely solely on a quantitative threshold with an arbitrary *P*-value of <.05. Furthermore, the small sample size for this intervention contributed to inadequate power to detect differences in these 3 categories and might be due to exclusion of a good portion of the respondents.^12^^,^^13^

Findings indicate increased knowledge among frontline HCWs in all 3 competencies in the 3 months during which DHMTs implemented capacity-building interventions.

The 2YL intervention was custom designed for specific performance problems in the 3 regions, namely lack of knowledge on EPI policy, inconsistent defaulter tracing, poor documentation of immunization data, and weak communication about vaccination with caregivers. “Success” for training interventions is determined by behavior change that leads to better outcomes and solves the performance problem. The evidence of knowledge growth as shown in our quantitative results is supported by many of the qualitative comments from HCWs indicating positive behavior changes following the capacity-building interventions. One interesting finding was the combination of improved knowledge regarding the simultaneous injection policy (i.e., MCV2 and Men A can be given simultaneously) and improved defaulter tracing as seen in field observations and follow-up with HCWs after training. Moreover, although communication seemed to have the least improved score, HCWs reported spending more time communicating with caregivers about vaccination and addressing their concerns. With continued mentorship, peer-to-peer learning, and skill building through various means (e.g., supportive supervision visits or a buddy system), communication between nurses and caregivers will continue to improve over time. These improvements would lead to fewer missed opportunities for vaccination during the 2YL by capturing more unvaccinated children than in the past and ensuring they receive all appropriate vaccinations at the same immunization visit. These practices prevent an accumulation of susceptible (unvaccinated) children who could potentially sustain disease transmission or cause outbreaks of vaccine-preventable diseases.

The increase of knowledge and self-reported improved behavior changes suggest that various interventions used by the DHMTs to reach HCWs may be promising practices for improving workforce performance. This includes follow-up and postintervention activities, such as supportive supervision visits, on-the-job training, and data review meetings to facilitate learning.^14^^,^^15^ Changes in practice and knowledge of HCWs could also be due to the effect of spacing learning, whereby the frontline HCWs engaged with these learning materials through a practical application over time in their health facilities.^16^ The DHMTs learned how to train HCWs using a learner-focused (i.e., based on expected job duties) and performance-based approach with embedded adult learning principles. The DHMTs used a variety of teaching methods and delivery techniques to facilitate learning, including the application of performance improvement strategies (e.g., group problem analysis, supervisory and interpersonal skills) to understand the underlying causes of workforce performance issues related to EPI identified within the district and health facility levels.^15^^–^^18^ These factors minimize the forgetting curve for the HCWs: learners tend to forget information learned during training if there are no efforts to apply acquired skills and knowledge.^19^^,^^20^

Increased knowledge and improved behavior changes suggest that interventions used by DHMTs to reach HCWs may be promising practices for improving workforce performance.

According to Gilbert’s^21^ behavior engineering model, clear expectations such as those provided by standard operating procedures and performance feedback have a bigger impact on workforce performance and health systems than improving knowledge. Gilbert’s^21^ model also demonstrates that providing HCWs with the necessary tools has a greater effect on their performance than knowledge and skill. We observed that some workers did not have the tools to record immunization data and had to improvise. While partner agencies facilitated the acquisition of CWC registers with subsequent training interventions for this project, for the long term we recommend that the district staff routinely assess whether subdistrict and health facility staff have a standard set of tools, keep track of the availability of these tools, and report to the regional or national staff about these needs. The national and regional EPI budget should include funds for printing and distribution of these materials to enable national EPI staff to provide these tools to the subdistrict and health facility staff. Lastly, based on field observations and study results some HCWs did not trace defaulters, know their target population for immunization, or master the catch-up policies for missed immunizations and simultaneous injections. This finding is a major concern because every health encounter should be used to identify and reach children with recommended vaccines, especially those who may have missed doses.^22^ GHS should leverage existing mechanisms, such as new hire orientation, supportive supervision visits, and monthly data review meetings to empower their HCWs to perform EPI tasks more proficiently. The ownership and continued attention by national and subnational leaders on the capacity-building needs of the immunization workforce will be key to ensure optimal performance of the immunization program. HCWs in Ghana and beyond could benefit from standardized simple quality job aids for immunization topics that could be translated into many local languages.

### Limitations

Due to budget limitations, a small sample was used for this study and focused on just a subset of 5 poorly performing districts in each of the 3 participating regions in the country. The small sample size did not allow for us to conduct multiple comparisons of subgroups and limited the power of the study to detect statistically significant increases in knowledge before and after the intervention. In addition, PAS1 was not administered to a substantial portion of eligible HCWs at the right time, so their exclusion from the analysis further reduced the sample size. Because of the restricted geographic focus, our results are not generalizable for the entire population in Ghana, and we do not know whether these interventions would yield similar results in other districts. The knowledge score of the HCWs could have been influenced by selection and social desirability biases—the data collectors could have unconsciously or consciously scored certain participants higher to be viewed favorably. Furthermore, all data on training quality and behavior change were self-reported and had the potential for recall and personal biases.

Also, study findings were not compared with district-level immunization performance data to verify the degree of performance improvement after the intervention among key immunization indicators (e.g., dropout rates, coverage). A 2YL endline survey is planned to evaluate the overall impact of various 2YL interventions (including workforce development) on improving immunization coverage and reducing variation in coverage equity among districts surveyed during the 2YL 2016 baseline assessment. This endline survey will address this data gap.

## CONCLUSION

This evaluation offers preliminary encouraging results in taking the first step toward improving HCW knowledge, attitudes, and practices for 3 core immunization competency areas. Following the 2YL interventions we have outlined, HCWs reported an overall increase in knowledge of the EPI policy; the importance of data validation/data review meetings to improve data management, recording, and use; the significance of conducting defaulter tracing; and the need to improve communication with caregivers about vaccination. HCWs also showed improved attitudes and practices in all 3 competency areas. The use of learner-focused teaching methods combined with adult learning principles was helpful in solving specific performance problems (such as lack of knowledge of EPI policy and poor documentation of data) and should be included as a standard practice for future training interventions. The upcoming 2YL endline survey will offer insights into the impact of this workforce development approach for improving immunization coverage in the target geographies.

## Notes

**Peer Reviewed**
